# Center-Level Procedure Volume Does Not Predict Failure-to-Rescue After Severe Complications of Oncologic Colon and Rectal Surgery

**DOI:** 10.1007/s00268-021-06296-w

**Published:** 2021-08-27

**Authors:** Miriam Lillo-Felipe, Rebecka Ahl Hulme, Maximilian Peter Forssten, Gary A. Bass, Yang Cao, Peter Matthiessen, Shahin Mohseni

**Affiliations:** 1 Department of Surgery, Orebro University Hospital, Orebro, Sweden; 2 Division of Trauma and Emergency Surgery, Department of Surgery, Karolinska University Hospital, Stockholm, Sweden; 3 School of Medical Sciences, Orebro University, Orebro, Sweden; 4 Division of Surgery, Department of Clinical Science, Intervention and Technology, Karolinska Institutet, Stockholm, Sweden; 5 Division of Traumatology, Surgical Critical Care & Emergency Surgery, University of Pennsylvania, Penn Presbyterian Medical Center, Philadelphia, USA; 6 Clinical Epidemiology and Biostatistics, School of Medical Sciences, Orebro University, Orebro, Sweden; 7 Division of Trauma and Emergency Surgery, Department of Surgery, Orebro University Hospital, 701 85 Orebro, Sweden

## Abstract

**Background:**

The relationship between hospital surgical volume and outcome after colorectal cancer surgery has thoroughly been studied. However, few studies have assessed hospital surgical volume and failure-to-rescue (FTR) after colon and rectal cancer surgery. The aim of the current study is to evaluate FTR following colorectal cancer surgery between clinics based on procedure volume.

**Methods:**

Patients undergoing colorectal cancer surgery in Sweden from January 2015 to January 2020 were recruited through the Swedish Colorectal Cancer Registry. The primary endpoint was FTR, defined as the proportion of patients with 30-day mortality after severe postoperative complications in colorectal cancer surgery. Severe postoperative complications were defined as Clavien–Dindo ≥ 3. FTR incidence rate ratios (IRR) were calculated comparing center volume stratified in low-volume (≤ 200 cases/year) and high-volume centers (> 200 cases/year), as well as with an alternative stratification comparing low-volume (< 50 cases/year), medium-volume (50–150 cases/year) and high-volume centers (> 150 cases/year).

**Results:**

A total of 23,351 patients were included in this study, of whom 2964 suffered severe postoperative complication(s). Adjusted IRR showed no significant differences between high- and low-volume centers with an IRR of 0.97 (0.75–1.26, *p* = 0.844) in high-volume centers in the first stratification and an IRR of 2.06 (0.80–5.31, *p* = 0.134) for high-volume centers and 2.15 (0.83–5.56, *p* = 0.116) for medium-volume centers in the second stratification.

**Conclusion:**

This nationwide retrospectively analyzed cohort study fails to demonstrate a significant association between hospital surgical volume and FTR after colorectal cancer surgery. Future studies should explore alternative characteristics and their correlation with FTR to identify possible interventions for the improvement of quality of care after colorectal cancer surgery.

## Introduction

The association between postoperative outcomes after cancer surgery and center volumes has been the topic of much debate, and the assumption that centers with higher caseload perform advanced operations with improved outcomes has driven centralization [1]. Several studies have associated this centralization of complex oncologic resections, including esophagectomy, pancreatectomy, liver resection, and pulmonary lobectomy, with improved outcomes [2–4]. Equipoise remains, however, in colorectal cancer surgery, with several studies demonstrating improvements in overall survival for high-volume centers [5–7], while other studies failed to detect any morbidity or mortality difference with increased procedure volume [8–11].

Failure-to-rescue (FTR), an emerging surgical outcome metric reflecting center-level quality of postoperative care, is described as "*the mortality rate among patients with complications*"[12]. Literature on center volume and its effect on FTR after colorectal cancer surgery is scarce. Some studies have shown that higher-volume centers (HVCs) have lower FTR rates than low-volume centers (LVCs), indicating better quality of care [13, 14], while others have not been able to do so [15, 16].

In a recently published study by our group, we noted a strong association between university hospital status and decreased FTR in colorectal cancer surgery across Sweden [17]. In this study, we aim to evaluate the effects of procedure volume on FTR in colorectal cancer surgery in the same study cohort. We hypothesize that HVCs will show higher survival rates after severe postoperative complications compared to LVCs.

## Material and methods

### Patient selection

Ethical approval was granted by the Swedish Ethics Review Authority (reference 2020-01622). This study complies with the principles of the Declaration of Helsinki and the STROBE guidelines (Supplementary Table) [18]. All patients who underwent colorectal cancer surgery from January 1, 2015, to January 1, 2020, in Sweden were retrieved from the prospectively accrued national Swedish Colorectal Cancer Registry (SCRCR). SCRCR has recently been validated to have high quality, with data completeness of > 98% [19]. Retrieved data included demographics, surgical and oncological treatments, and outcomes for all patients undergoing colorectal cancer surgery within the study period. Only colorectal cancer resections were included. Severe complications were defined according to Clavien-Dindo as a score of ≥ 3[20]. The primary outcome of interest was failure-to-rescue, defined in accordance with previous FTR studies [16, 21], as the ratio of patients with a severe complication who died within 30 days of surgery to the total number of patients with a severe complication.

## Statistical analysis

Centers were divided into cohorts based on their procedure volumes, where hospitals with an average of ≤ 200 cases/year were classified as low volume and hospitals with an average of > 200 cases/year were classified as high volume [15]. An alternative stratification method was also used with LVCs defined as < 50 cases/year, medium-volume centers (MVCs) between 50 and 150 cases/year, and HVCs > 150 cases/year.

Categorical variables between the cohorts were compared using the Chi-square test or Fisher's exact test, while the Student's *t*-test was used for normally distributed continuous variables. The Mann–Whitney U test was used for non-normally distributed continuous variables. A Poisson regression model with robust standard errors of variance was carried out for calculating the incidence risk ratios (IRR) for FTR. For each stratification method, a univariable and multivariable model was calculated. The multivariable Poisson regressions were adjusted for age, sex, body mass index (BMI), American Society of Anesthesiologists (ASA) classification, cancer stage, surgical technique (minimally invasive or open surgery), type of surgery, and unplanned reoperation.

## Results

A total of 23,351 patients underwent colorectal cancer surgery in Sweden during the five-year inclusion period. Patient demographics for the entire cohort are presented in Table 1. Depicted in Table 2 are demographics of patient who suffered a severe complication. The majority of patients (*n* = 16,943, 72.6%) underwent surgery for colon cancer. Eighty-nine percent of operations were elective cases. Most cases were performed using open rather than a minimally invasive surgery (57.6% vs. 42.3%). A total of 6,463 patients (27.7%) suffered some form of complication, and 2,964 patients (12.7%) had a Clavien–Dindo score of ≥ 3 (Table 1).

**Table 1:** Demographics of patients stratified by hospital caseload, according to Henneman et al.

	Low volume (≤ 200 cases/year) (*N* = 14,246)	High volume (> 200 cases/year) *(N* = 9,105)	*P*-value
*Age in years*			
Mean (SD)	72.0 (± 11.0)	70.3 (± 11.7)	< 0.001
Median [IQR]	73 [66–80]	72 [64–79]	< 0.001
BMI			
Mean (SD)	26.2 (± 4.6)	26.1 (± 4.7)	0.175
Median [IQR]	26 [23–29]	26 [23–29]	0.085
Missing, n (%)	6026 (42.3)	4151 (45.6)	
Sex, n (%)			0.350
Female	6809 (47.8)	4294 (47.2)	
Male	7437 (52.2)	4811 (52.8)	
ASA classification, n (%)			< 0.001
1	1624 (11.4)	1035 (11.4)	
2	7507 (52.7)	4368 (48.0)	
3	4380 (30.7)	3213 (35.3)	
4	348 (2.4)	305 (3.3)	
5	9 (0.1)	6 (0.1)	
Missing	378 (2.7)	178 (2.0)	
Cancer stage, n (%)			< 0.001
1	3047 (21.4)	1858 (20.4)	
2	4895 (34.4)	2888 (31.7)	
3	4778 (33.5)	3203 (35.2)	
4	1038 (7.3)	734 (8.1)	
Missing	488 (3.4)	422 (4.6)	
Tumor location, n (%)			< 0.001
Colon	10561 (74.1)	6382 (70.1)	
Rectum	3685 (25.9)	2723 (29.9)	
Type of surgery, n (%)			< 0.001
Ileocecal resection	91 (0.6)	57 (0.6)	
Right hemicolectomy	5859 (41.1)	3370 (37.0)	
Transverse colon resection	167 (1.2)	117 (1.3)	
Left hemicolectomy	1069 (7.5)	678 (7.4)	
Sigmoid colon resection	2193 (15.4)	1312 (14.4)	
Total colectomy	534 (3.7)	405 (4.4)	
Anterior resection	2157 (15.1)	1560 (17.1)	
Abdomino-perineal excision	1399 (9.8)	1088 (11.9)	
Hartmann's operation	777 (5.5)	518 (5.7)	
Neoadjuvant therapy, n (%)	1005 (7.1)	938 (10.3)	< 0.001
Missing	73 (0.5)	42 (0.5)	
Curative treatment, n (%)	12773 (89.7)	8175 (89.8)	0.775
Surgical setting, n (%)			0.008
Elective	12762 (89.6)	8057 (88.5)	
Acute	1477 (10.4)	1046 (11.5)	
Missing	7 (0.0)	2 (0.0)	
Minimally invasive surgery, n (%)	5867 (41.2)	4013 (44.1)	< 0.001
Missing	25 (0.2)	7 (0.1)	
Reoperation, n (%)	1218 (8.5)	718 (7.9)	0.140
Missing	323 (2.3)	325 (3.6)	
Length of stay, median [IQR]	7.0 [4.0–10]	7.0 [4.0–12]	< 0.001
Missing, n (%)	395 (2.8)	396 (4.3)	
Length of ICU stay, median [IQR]	3.0 [1.0–6.0]	2.0 [1.0–5.0]	0.110
Missing, n (%)	13533 (95.0)	8742 (96.0)	
30-day mortality, n (%)	230 (1.6)	148 (1.6)	0.991
Any complication, n (%)	3527 (24.8)	2936 (32.2)	< 0.001
*Severe complication, n (%)*	*1754 (12.3)*	*1210 (13.3)*	*0.030*

*SD*, standard deviation; *IQR*, interquartile range; *BMI*, body mass index; *ASA*, American Society of Anesthesiologists; *ICU*, intensive care unit

**Table 2:** Demographics of patients with severe complications stratified by hospital caseload, according to Henneman et al.

	Low volume (≤ 200 cases/year) (*N* = 1754)	High volume (> 200 cases/year) (*N* = 1210)	*P*-value
*Age in years*			
Mean (SD)	72.1 (± 10.5)	69.8 (± 11.8)	< 0.001
Median [IQR]	73 [66–80]	72 [64–78]	< 0.001
BMI			
Mean (SD)	26.7 (± 5.0)	26.5 (± 5.3)	0.438
Median [IQR]	26 [24–29]	26 [23–29]	0.453
Missing, n (%)	789 (45.0)	584 (48.3)	
Sex, n (%)			0.781
Female	664 (37.9)	465 (38.4)	
Male	1090 (62.1)	745 (61.6)	
ASA classification, n (%)			0.006
1	158 (9.0)	118 (9.8)	
2	833 (47.5)	506 (41.8)	
3	638 (36.4)	482 (39.8)	
4	69 (3.9)	70 (5.8)	
5	2 (0.1)	1 (0.1)	
Missing	54 (3.1)	33 (2.7)	
Cancer stage, n (%)			< 0.001
1	357 (20.4)	203 (16.8)	
2	621 (35.4)	376 (31.1)	
3	596 (34.0)	436 (36.0)	
4	135 (7.7)	147 (12.1)	
Missing	45 (2.6)	48 (4.0)	
Tumor location, n (%)			< 0.001
Colon	1205 (68.7)	726 (60.0)	
Rectum	549 (31.3)	484 (40.0)	
Type of surgery, n (%)			< 0.001
Ileocecal resection	10 (0.6)	14 (1.2)	
Right hemicolectomy	611 (34.8)	340 (28.1)	
Transverse colon resection	19 (1.1)	14 (1.2)	
Left hemicolectomy	145 (8.3)	98 (8.1)	
Sigmoid colon resection	241 (13.7)	128 (10.6)	
Total colectomy	125 (7.1)	76 (6.3)	
Anterior resection	293 (16.7)	274 (22.6)	
Abdomino-perineal excision	190 (10.8)	182 (15.0)	
Hartmann's operation	120 (6.8)	84 (6.9)	
Neoadjuvant therapy, n (%)	146 (8.3)	167 (13.8)	< 0.001
Missing	9 (0.5)	8 (0.7)	
Curative treatment, n (%)	1535 (87.5)	1036 (85.6)	0.150
Surgical setting, n (%)			0.038
Elective	1523 (86.8)	1017 (84.0)	
Acute	231 (13.2)	193 (16.0)	
Minimally invasive surgery, n (%)	588 (33.5)	452 (37.4)	0.035
Missing	2 (0.1)	1 (0.1)	
Reoperation, n (%)	1118 (63.7)	679 (56.1)	< 0.001
Length of stay, median [IQR]	15 [8.0–23]	15 [9.0–24]	0.112
Missing, n (%)	13 (0.7)	18 (1.5)	
Length of ICU stay, median [IQR]	4.0 [2.0–8.0]	3.0 [2.0–6.0]	0.012
30-day mortality, n (%)	171 (9.7)	114 (9.4)	0.815

*SD*, standard deviation; *IQR*, interquartile range; *BMI*, body mass index; *ASA*, American Society of Anesthesiologists; *ICU*, intensive care unit

When centers were stratified using a 200 case/year threshold, patients were older in LVCs [mean age (SD): 72 (11) vs. 70 (12) years, *p* < 0.001]. HVCs treated a greater proportion of patients with stage 3–4 cancer (43.3% vs. 40.8%, *p* < 0.001) compared to LVCs. A significantly higher percentage of patients with rectal cancer were operated in HVCs than LVCs (29.9% vs. 25.9%, *p* < 0.001), congruent with a national strategy favoring volume-cohorting of rectal cancers. Crude results showed that HVCs had higher rates of overall complications compared to LVCs (32.2% vs. 24.8%, *p* < 0.001), as well as severe complications (13.3% vs. 12.3%, *p* = 0.03). Despite this, 30-day mortality did not differ between high-volume and low-volume hospitals (1.6%. vs. 1.6%, *p* = 0.991). There were also no statistically significant differences in unplanned reoperations or ICU length of stay (LOS) between HVCs and LVCs (Table 1). Patients with severe complications were older in LVCs. Most severe postoperative complications occurred after colon cancer surgery, corresponding to 68.7% of all severe complications in LVCs compared to 60.0% in HVCs. Reoperations after severe complications were required more frequently in LVCs than HVCs, with a reoperation frequency of 63.7% and 56.1%, respectively. There was no significant difference in 30-day mortality rates for patients with severe complications (9.7% vs. 9.4%, *p* = 0.815) (Table 2).

**Fig. 1:**
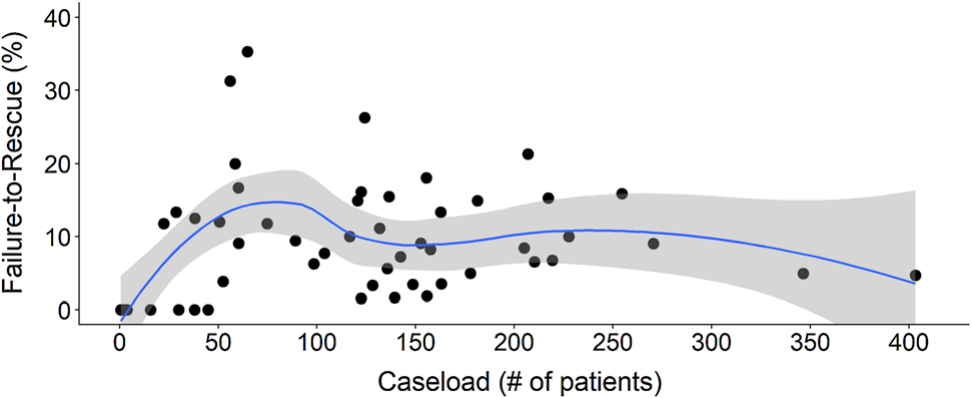
Mortality rate (%) among patients with severe complications (Clavien-Dindo ≥ 3) per colorectal unit according to their average yearly procedure volume

When classified according to our alternative 3-level stratification method, there was a significant decrease in the proportion of patients with stage 3–4 cancer when comparing low-, medium- and high-volume centers (42.3%, vs. 41.9% vs. 41.7%, *p* = 0.010). Compared to LVCs, medium- and HVCs had higher proportions of patients with rectal cancer (10.5% vs. 29.4% and 25.8%, *p* < 0.001). Unplanned reoperations were more common in LVCs compared to medium- and HVCs (12.6% vs. 8.7% and 7.8%, *p* < 0.001). Crude results showed that HVCs and LVCs had higher rates of overall complications compared to medium-volume centers (29.4% and 28.4% vs. 24.7%, p < 0.001). There was no significant difference in severe complications (14.4%, 12.8%, 12.5%, *p* = 0.256) or 30-day crude mortality (1.5% vs. 1.7% vs. 1.8%, *p* = 0.498) between centers (Table 3). When comparing the stratified volume groups, the mean age for patients with severe complications was higher in low- and medium-volume centers than HVCs. Most severe postoperative complications occurred after colon cancer surgery, corresponding to 87.5% of all severe complications in LVCs compared to 68.3% and 61.7% in medium- and HVCs, respectively. Reoperations were required more frequently in LVCs than medium- and HVCs, (81.7% compared to 61.5% and 58.6%, respectively, *p* = 0.010). There was no significant difference in 30-day mortality rates for patients with severe complications (5.8% vs. 10.3% vs. 9.4%, *p* = 0.269) (Table 4). Figure 1 shows the association between mortality following a severe complication and procedure volume and demonstrates no correlation.

**Table 3:** Demographics of patients stratified by hospital caseload, according to the alternative stratification

	Low volume (< 50 cases/year) (*N* = 831)	Medium volume (50–150 cases/year) (*N* = 8494)	High volume (> 150 cases/year) (*N* = 14,026)	*P*-value
*Age in years*				
Mean (SD)	73.0 (± 10.7)	72.0 (± 10.8)	70.8 (± 11.6)	< 0.001
Median [IQR]	74 [67–81]	73 [66–80]	72 [64–79]	< 0.001
*BMI*				
Mean (SD)	26.2 (± 4.7)	26.3 (± 4.6)	26.1 (± 4.7)	0.073
Median [IQR]	26 [23–29]	26 [23–29]	26 [23–29]	0.056
Missing, n (%)	373 (44.9)	3594 (42.3)	6210 (44.3)	
Sex, n (%)				0.413
Female	413 (49.7)	4017 (47.3)	6673 (47.6)	
Male	418 (50.3)	4477 (52.7)	7353 (52.4)	
ASA classification, n (%)				< 0.001
1	74 (8.9)	959 (11.3)	1626 (11.6)	
2	373 (44.9)	4576 (53.9)	6926 (49.4)	
3	338 (40.7)	2530 (29.8)	4725 (33.7)	
4	15 (1.8)	208 (2.4)	430 (3.1)	
5	0 (0.0)	6 (0.1)	9 (0.1)	
Missing	31 (3.7)	215 (2.5)	310 (2.2)	
Cancer stage, n (%)				0.010
1	152 (18.3)	1731 (20.4)	3022 (21.5)	
2	306 (36.8)	2933 (34.5)	4544 (32.4)	
3	291 (35.0)	2894 (34.1)	4796 (34.2)	
4	61 (7.3)	662 (7.8)	1049 (7.5)	
Missing	21 (2.5)	274 (3.2)	615 (4.4)	
Tumor location, n (%)				< 0.001
Colon	744 (89.5)	6301 (74.2)	9898 (70.6)	
Rectum	87 (10.5)	2193 (25.8)	4128 (29.4)	
Type of surgery, n (%)				< 0.001
Ileocecal resection	9 (1.1)	55 (0.6)	84 (0.6)	
Right hemicolectomy	419 (50.4)	3513 (41.4)	5297 (37.8)	
Transverse colon resection	6 (0.7)	93 (1.1)	185 (1.3)	
Left hemicolectomy	84 (10.1)	668 (7.9)	995 (7.1)	
Sigmoid colon resection	167 (20.1)	1322 (15.6)	2016 (14.4)	
Total colectomy	29 (3.5)	323 (3.8)	587 (4.2)	
Anterior resection	53 (6.4)	1144 (13.5)	2520 (18.0)	
Abdomino-perineal excision	37 (4.5)	870 (10.2)	1580 (11.3)	
Hartmann's operation	27 (3.2)	506 (6.0)	762 (5.4)	
Neoadjuvant therapy, n (%)	41 (4.9)	516 (6.1)	1386 (9.9)	< 0.001
Missing	5 (0.6)	26 (0.3)	84 (0.6)	
Curative treatment, n (%)	744 (89.5)	7520 (88.5)	12684 (90.4)	< 0.001
Surgical setting, n (%)				0.668
Elective	733 (88.2)	7582 (89.3)	12504 (89.1)	
Acute	97 (11.7)	907 (10.7)	1519 (10.8)	
Missing	1 (0.1)	5 (0.1)	3 (0.0)	
Minimally invasive surgery, n (%)	381 (45.8)	3314 (39.0)	6185 (44.1)	< 0.001
Missing	3 (0.4)	18 (0.2)	11 (0.1)	
Reoperation, n (%)	105 (12.6)	742 (8.7)	1089 (7.8)	< 0.001
Missing	33 (4.0)	214 (2.5)	401 (2.9)	
Length of stay, median [IQR]	6.0 [4.0–10]	6.0 [4.0–10]	7.0 [4.0–11]	< 0.001
Missing, n (%)	36 (4.3)	262 (3.1)	493 (3.5)	
Length of ICU stay, median [IQR]	3.0 [2.0–5.0]	3.0 [1.0–6.0]	2.0 [1.0–5.0]	0.135
Missing, n (%)	774 (93.1)	8052 (94.8)	13449 (95.9)	
30-day mortality, n (%)	15 (1.8)	147 (1.7)	216 (1.5)	0.498
Any complication, n (%)	236 (28.4)	2097 (24.7)	4130 (29.4)	< 0.001
Severe complication, n (%)	120 (14.4)	1087 (12.8)	1757 (12.5)	0.256

*SD*, standard deviation; *IQR*, interquartile range; *BMI*, body mass index; *ASA*, American Society of Anesthesiologists; *ICU*, intensive care unit

**Table 4:** Demographics of patients with severe complications stratified by hospital caseload, according to the alternative stratification

	Low volume (< 50 cases/year) (*N* = 120)	Medium volume (50–150 cases/year) (*N* = 1,087)	High volume (> 150 cases/year) (*N* = 1,757)	*P*-value
*Age in years*				
Mean (SD)	72.4 (± 11.4)	72.4 (± 10.1)	70.3 (± 11.6)	< 0.001
Median [IQR]	74 [67–81]	73 [67–80]	72 [64–78]	< 0.001
*BMI*				
Mean (SD)	27.7 (± 5.9)	26.7 (± 4.9)	26.5 (± 5.1)	0.187
Median [IQR]	28 [23–31]	26 [24–29]	26 [23–29]	0.321
Missing, n (%)	54 (45.0)	484 (44.5)	835 (47.5)	
Sex, n (%)				0.549
Female	40 (33.3)	416 (38.3)	673 (38.3)	
Male	80 (66.7)	671 (61.7)	1084 (61.7)	
ASA classification, n (%)				< 0.001
1	3 (2.5)	103 (9.5)	170 (9.7)	
2	43 (35.8)	523 (48.1)	773 (44.0)	
3	70 (58.3)	378 (34.8)	672 (38.2)	
4	1 (0.8)	47 (4.3)	91 (5.2)	
5	0 (0.0)	2 (0.2)	1 (0.1)	
Missing	3 (2.5)	34 (3.1)	50 (2.8)	
Cancer stage, n (%)				0.114
1	23 (19.2)	211 (19.4)	326 (18.6)	
2	47 (39.2)	384 (35.3)	566 (32.2)	
3	41 (34.2)	383 (35.2)	608 (34.6)	
4	8 (6.7)	85 (7.8)	189 (10.8)	
Missing	1 (0.8)	24 (2.2)	68 (3.9)	
Tumor location, n (%)				< 0.001
Colon	105 (87.5)	742 (68.3)	1084 (61.7)	
Rectum	15 (12.5)	345 (31.7)	673 (38.3)	
Type of surgery, n (%)				< 0.001
Ileocecal resection	1 (0.8)	5 (0.5)	18 (1.0)	
Right hemicolectomy	46 (38.3)	396 (36.4)	509 (29.0)	
Transverse colon resection	1 (0.8)	10 (0.9)	22 (1.3)	
Left hemicolectomy	19 (15.8)	89 (8.2)	135 (7.7)	
Sigmoid colon resection	23 (19.2)	137 (12.6)	209 (11.9)	
Total colectomy	11 (9.2)	75 (6.9)	115 (6.5)	
Anterior resection	10 (8.3)	169 (15.5)	388 (22.1)	
Abdomino-perineal excision	4 (3.3)	127 (11.7)	241 (13.7)	
Hartmann's operation	5 (4.2)	79 (7.3)	120 (6.8)	
Neoadjuvant therapy, n (%)	5 (4.2)	77 (7.1)	231 (13.1)	< 0.001
Missing	0 (0.0)	5 (0.5)	12 (0.7)	
Curative treatment, n (%)	107 (89.2)	938 (86.3)	1526 (86.9)	0.663
Surgical setting, n (%)				0.847
Elective	101 (84.2)	935 (86.0)	1504 (85.6)	
Acute	19 (15.8)	152 (14.0)	253 (14.4)	
Minimally invasive surgery, n (%)	41 (34.2)	372 (34.2)	627 (35.7)	0.709
Missing	0 (0.0)	1 (0.1)	2 (0.1)	
Reoperation, n (%)	98 (81.7)	669 (61.5)	1030 (58.6)	< 0.001
Length of stay, median [IQR]	14 [9.0–23]	14 [8.0–24]	16 [9.0–24]	0.054
Missing, n (%)	0 (0.0)	9 (0.8)	22 (1.3)	
Length of ICU stay, median [IQR]	4.0 [2.0–8.0]	4.0 [2.0–8.0]	3.0 [2.0–7.0]	0.213
30-day mortality, n (%)	7 (5.8)	112 (10.3)	166 (9.4)	0.269

*SD, *standard deviation;* IQR, *interquartile range*; BMI, *body mass index*; ASA, *American Society of Anesthesiologists;* ICU, *intensive care unit

A multivariable analysis of FTR and hospital procedure volume is outlined in Table 5. Irrespective of the chosen stratification method, the risk of FTR was not statistically different in any of the caseload groups. This finding remained unchanged in both univariable and multivariable analyses.

**Table 5:** Incidence rate ratio for FTR and hospital caseload

	Unadjusted FTR* (%)	Unadjusted FTR IRR (95% CI)	P-value	Adjusted FTR IRR (95% CI)	*P*-value
*Hospital caseload* *(2 groups)*					
Low (≤ 200 cases/year)	10%	reference		reference	
High (> 200cases/year)	9%	0.96 (0.75–1.24)	0.79	0.97 (0.75–1.26)	0.844
*Hospital caseload* *(3 groups)*					
Low (< 50 cases/year)	6%	reference		reference	
Medium (50–150 cases/year)	10%	1.81 (0.79–4.12)	0.159	2.15 (0.83–5.56)	0.116
High (> 150 cases/year)	9%	1.65 (0.73–3.73)	0.231	2.06 (0.80–5.31)	0.134

Poisson regression with robust standard errors of variance. Missing values were managed using multiple imputation by chained equations. All models were adjusted for age, sex, body mass index, American Society of Anesthesiologists classification, cancer stage, surgical technique, type of surgery, and unplanned reoperation

^*^ FTR = 30-day mortality among patients with severe complications/total amount of severe complications

*FTR, *failure-to-rescue;* IRR, *incident rate ratio;* CI, *confidence interval

## Discussion

In this large national observational cohort study, centers with high procedure volumes (HVCs) did not have significantly lower FTR rates than centers with lower case volumes. Centers were stratified in two different ways by their procedure volume; however, no correlation was found between volume and FTR.

Center volume, as a surrogate marker for quality, has long been considered one of several factors related to FTR after major surgery [22]. Other variables associated with lower postoperative mortality and FTR include academic teaching status [17], specialist nurse staffing, larger teams of senior doctors in surgical and medical specialties, structured transitions of care[23], advanced technology and treatment strategies in clinical practice [22, 24–26]. In the past, HVCs have been pioneers in developing efficient and safe pathways to improve logistics and surgical results. Hospitals with lower caseloads have subsequently adopted many of these protocols, which are now almost ubiquitous in modern colorectal cancer units, regardless of surgical volume. Prompt detection and amelioration of potentially severe complications by the surgical and perioperative nursing teams is necessary to improve FTR rates [24].

The results of the current study fail to demonstrate association between procedure volume and FTR of severe postoperative complications. Previous analysis of the same cohort (stratified by University Hospital status) showed a 38% (adjusted IRR 0.62, 95% CI 0.46–0.84, *p* = 0.002) decrease in FTR in University hospitals [17]. This apparent incongruity may in fact suggest that resource availability, such as round-the-clock radiology and dedicated ICUs, exerts a greater impact on survival after severe postoperative complications than a center’s raw caseload.

Interestingly, the LOS in HVCs was longer than lower- and medium-volume centers. This may partly be due to HVC case mix, having a higher proportion of rectal cancer surgery with high complication risks. LOS is an important surrogate marker for quality of care and treatment success following major surgery [27]. Vicendese et al. were unable to demonstrate that LOS was affected by procedure volume and postulated that other factors are of greater importance for clinical outcomes [28].

Although high procedure volume does not appear to improve FTR, many agree there should be a minimum requirement for procedure volumes in the context of cancer surgery to achieve better oncological outcomes. This idea is supported by a newly published study from Diers et al., who found up to 33% lower mortality and FTR rates after colorectal cancer surgery in centers achieving the minimum set at 30 cases per year for colon cancer and 20 cases per year for rectal cancer [14]. In future studies, FTR may be used to establish a benchmark, beyond procedure volumes and postoperative complications or oncological outcomes, for colorectal units.

Our study has several strengths and weaknesses. Data come from the SCRC registry, covering > 98% of all colorectal cancer operations in Sweden. Nonetheless, caution should be exercised when assessing postoperative complications using the SCRC registry. A recent validation study from Moberger et al. showed that complications data showed exact database agreement of only 89% for surgical complications and 84% for medical complications. In contrast, postoperative course and follow-up had an exact agreement in 98% [19]. However, including severe complications only when calculating FTR makes the accuracy of the complications data more reliable. Additionally, this is the first study to investigate FTR and procedure volume after colorectal cancer surgery in Sweden. The study aimed to assess the influence of center-level procedure volume on the ability to detect and recover severe complications and not the absolute risk of such adverse events per se. Thus, separate analyses for colon and rectal cancers were not performed, which is in line with previous studies [15, 16, 29].

There are several limitations to the current study. Firstly, this is a retrospective analysis of prospectively collected data, and individual assessments of FTR cannot be made. It would, however, be ethically challenging to address FTR and its association with procedure volume in randomized controlled trials. Secondly, in the current study we do not include surgeon-specific procedure volume nor surgical experience. Such factors may have an important impact on the peri- and postoperative outcomes but do not necessarily have a direct effect on FTR, since FTR is a metric used to evaluate the whole postoperative pathway and not the individual surgeon’s performance. Currently, there is no data specifically examining the association between characteristics of the operating surgeon and FTR [24].

One possible confounder that we were unable to control was that of possible inter-hospital transfers in case of complications. There are no transfer data available for analysis in the SCRCR. Due to a shortage of ICU beds in Sweden, inter-hospital transfers of critically ill patients between intensive care units have increased over the last few years. However, only 6% of hospital transfers affect elective surgery cases, thereby indicating that this possible confounder should have little effect [30].

It must be noted that results from the current study may not be generalizable to all healthcare systems, when considering the unique situation of Swedish colorectal cancer surgery, that has highly specialized colorectal surgeons performing the vast majority of colorectal procedures and centralized rectal cancer surgery. All 61 hospitals are considered “teaching hospitals” as they facilitate training for residents in general surgery. Nearly all rectal cancer and > 95% of colon cancer cases are discussed in multidisciplinary teams regardless of hospital volume [31]. Moreover, rectal cancer surgery in Sweden has also been centralized over the last 20 years to ensure adequate case volumes to safeguard surgical quality [32]. These aspects must be considered when assessing any causal relationship between procedure volume and FTR.

When assessing the volume-outcome relationship in colorectal cancer surgery, both Archampong et al. and Chioreso et al. found there to be a provider variability at hospital level between different countries, especially when comparing US and non-US data. They saw a significant volume–outcome relationship after colorectal cancer surgery in US-based studies but not in European and other non-US-based studies [1, 33]. Chioreso et al. stressed that this could in part be due to the degree of centralization of rectal cancer surgery outside the USA, and especially in Europe [33]. It is plausible to assume that the same may apply to procedure volume and FTR in colorectal cancer surgery. It is, therefore, important for each healthcare system to make their own assessment.

Another important limitation is the definition of low-, medium- and high-volume centers in different studies, which severely limits generalizability of results. The authors decided therefore to use two stratification methods to facilitate comparability with available literature. The cutoffs used in the current study (stratification using a 200 cases per year threshold) were the same used by Henneman et al., the first study to evaluate hospital characteristics in colorectal cancer surgery and FTR [15]. An alternative three-level stratification was also used by the authors, considering 50 resections/year to be the cutoff for the lowest volume and 150 resections/year for the highest volume.

Finally, centers are not differentiated by factors other than procedure volume. Access to other medical and surgical specialties play a vital role in dealing with some of the most common and deadly postoperative non-surgical complications such as myocardial infarction, pulmonary embolism, sepsis and stroke.

## Conclusion

This large nationwide retrospective cohort study fails to demonstrate a significant association between procedure volume and FTR after colorectal cancer surgery. It would be reasonable to deduce that characteristics other than center volume may have greater bearing on FTR rates. Therefore, we encourage future studies to explore alternative characteristics and their correlation with FTR, to identify possible interventions for the improvement of quality of care after colorectal cancer surgery.
